# New Supplemental Benefits and Plan Ratings Among Medicare Advantage Enrollees

**DOI:** 10.1001/jamanetworkopen.2024.15058

**Published:** 2024-06-05

**Authors:** Emma L. Tucher, David J. Meyers, Amal N. Trivedi, Laura M. Gottlieb, Kali S. Thomas

**Affiliations:** 1 Division of Research, Kaiser Permanente Northern California, Oakland; 2 Department of Health Services, Policy, and Practice, Brown University School of Public Health, Providence, Rhode Island; 3 Center for Gerontology and Health Care Research, Brown University School of Public Health, Providence, Rhode Island; 4 Providence Veterans Affairs Medical Center, Providence, Rhode Island; 5 Department of Family and Community Medicine, University of California, San Francisco; 6 Center for Equity in Aging, Johns Hopkins School of Nursing, Baltimore, Maryland

## Abstract

**Importance:**

In 2018, the US Congress gave Medicare Advantage (MA) historic flexibility to address members’ social needs with a set of Special Supplemental Benefits for the Chronically Ill (SSBCIs). In response, the Centers for Medicare & Medicaid Services expanded the definition of primarily health-related benefits (PHRBs) to include nonmedical services in 2019. Uptake has been modest; MA plans cited a lack of evidence as a limiting factor.

**Objective:**

To evaluate the association between adopting the expanded supplemental benefits designed to address MA enrollees’ nonmedical and social needs and enrollees’ plan ratings.

**Design, Setting, and Participants:**

This cohort study compared the plan ratings of MA enrollees in plans that adopted an expanded PHRB, SSBCI, or both using difference-in-differences estimators with MA Consumer Assessment of Health Care Providers and Systems survey data from March to June 2017, 2018, 2019, and 2021 linked to Medicare administrative claims and publicly available benefits and enrollment data. Data analysis was performed between April 2023 and March 2024.

**Exposure:**

Enrollees in MA plans that adopted a PHRB and/or SSBCI in 2021.

**Main Outcomes and Measures:**

Enrollee plan rating on a 0- to 10-point scale, with 0 indicating the worst health plan possible and 10 indicating the best health plan possible.

**Results:**

The study sample included 388 356 responses representing 467 MA contracts and 2558 plans in 2021. Within the weighted population of responders, the mean (SD) age was 74.6 (8.7) years, 57.2% were female, 8.9% were fully Medicare-Medicaid dual eligible, 74.6% had at least 1 chronic medical condition, 13.7% had not graduated high school, 9.7% were helped by a proxy, 45.1% reported fair or poor physical health, and 15.6% were entitled to Medicare due to disability. Adopting both a new PHRB and SSBCI benefit in 2021 was associated with an increase of 0.22 out of 10 points (95% CI, 0.4-4.0 points) in mean enrollee plan ratings. There was no association between adoption of only a PHRB (adjusted difference, −0.12 points; 95% CI, −0.26 to 0.02 points) or SSBCI (adjusted difference, 0.09 points; 95% CI, −0.03 to 0.21 points) and plan rating.

**Conclusions and Relevance:**

Medicare Advantage plans that adopted both benefits saw modest increases in mean enrollee plan ratings. This evidence suggests that more investments in supplemental benefits were associated with improved plan experiences, which could contribute to improved plan quality ratings.

## Key Points

**Question:**

Was Medicare Advantage plans’ adoption of supplemental benefits targeting members’ nonmedical and social needs associated with changes in enrollees’ ratings of plan experience?

**Findings:**

This cohort study compared 388 356 plan ratings of US Medicare Advantage enrollees in plans that adopted an expanded primarily health-related benefit, Special Supplemental Benefit for the Chronically Ill, or both and found that plans that adopted both benefits saw a significant mean increase of 0.22 points (of 10) in plan ratings vs plans that adopted neither.

**Meaning:**

These findings suggest that more investments in supplemental investments may improve plan ratings and experiences for medically and socially complex Medicare beneficiaries.

## Teaser

This cohort study of Medicare Advantage enrollees examines the association between adoption of a primarily health-related benefit, Special Supplemental Benefit for the Chronically Ill, or both and changes in enrollees’ overall plan ratings.

## Introduction

For older adults, social service investments, including housing support, nutrition assistance, and income support, are associated with health improvements and reductions in costly medical care.^1,2,3,4,5,6,7^ Medicare investments in these types of services were historically limited. However, in 2019, the Centers for Medicare & Medicaid Services (CMS) expanded the definition of primarily health-related benefits (PHRBs) from a set of medical services (eg, dental services, eyeglasses, and hearing aids) to allow Medicare Advantage (MA) plans to offer nonmedical services (eg, adult daycare, in-home support services, and caregiver support).^8^ Beginning in 2020, the US Congress allowed MA plans to address chronically ill members’ social needs through the Special Supplemental Benefits for the Chronically Ill (SSBCIs), which included more generous meals than those allowed previously, food and produce benefits, nonmedical transportation services, indoor air quality supports, and structural home modifications.^9^

Over time, the rate of supplemental benefit adoption has increased. By 2021, 19% of plans adopted a PHRB, an increase from 13% in 2019, and 17% of plans adopted an SSBCI, an increase from 5% in 2020.^8,9,10^ Early evidence suggests that members are using the newly covered services offered by plans. For instance, Elevance Health documented high use of the supplemental benefits: in 2022, 83% of Medicare and Medicaid dual-eligible and 75% of non–dual-eligible members used at least 1 new supplemental benefit, with 23% of dual-eligible and 14% of non–dual-eligible members using 4 or more.^11^ Despite increased adoption and use over time, there is no evidence of an association between plan experience and benefit adoption.

Medicare Advantage plans did not receive additional funding to invest in the supplemental benefits; instead, they are typically paid for through plan rebates and premiums.^12^ This contributes to a complex cost benefit analysis around providing expanded supplemental benefits. One rich source of information contributing to that analysis is member experience measures, such as overall health plan ratings. These measures are particularly valuable to plans because they are included in CMS Star Rating metrics, which influences annual bonus payments and rebates from CMS.^13^ Using the MA Consumer Assessments of Healthcare Providers and Systems (MA CAHPS) survey, a nationally representative, randomly sampled survey of community-dwelling MA members, this study examines the association between adoption of a PHRB, SSBCI, or both and changes in enrollees’ overall plan ratings.

## Methods

### Data and Measures

The MA CAHPS is a nationally representative, cross-sectional annual survey of MA enrollees. For this cohort study, we used data from the 2017, 2018, 2019, and 2021 MA CAHPS waves. Each January a sample of approximately 800 enrollees per contract was selected and surveyed between March and June. We linked these data to other Medicare sources, including the Medicare Beneficiary Summary File, for an enrollee’s plan and contract identifiers and Medicare and Medicaid dual eligibility status and then to publicly available plan benefit, crosswalk, and enrollment data. The plan benefit data provided information on supplemental benefit adoption, plan deductible, maximum out-of-pocket (OOP) spending, premium, and type. The crosswalk files linked plans across years and provided the Special Needs Plan (SNP) status. The monthly MA enrollment files provided plan- and county-level enrollment and the contract start date. We linked these data to county-level information on MA penetration using 5-digit Federal Information Processing Standards codes. This study was approved by the Brown University Institutional Review Board and received a waiver of informed consent due to the inability to contact enrollees from deidentified claims data. This study followed the Strengthening and Reporting of Observational Studies in Epidemiology (STROBE) reporting guidelines for cohort studies.

Our sample included all MA CAHPS respondents who completed the plan rating question (eFigure 1 in Supplement 1). The mean (SD) MA CAHPS response rate was 37.7% (0.5%) (n = 974 336 responses across waves). We included individuals enrolled in health maintenance organizations or preferred provider organizations. We excluded individuals in other plans (n = 245 807), who could not be linked to Medicare administrative data (n = 37), who did not respond to the plan rating question (n = 28 259), and who enrolled in plans that had adopted 1 or more supplemental benefits in 2019 or 2020 (n = 269 546). We matched based on an individual’s county of residence and excluded individuals from unmatched counties (n = 38 074).

The exposure of interest was whether an individual was enrolled in a plan that adopted a PHRB, SSBCI, or both benefits in 2021 that had not previously adopted a supplemental benefit (eTable 1 in Supplement 1). The PHRBs were adult daycare, home-based palliative care, in-home support services, caregiver supports, and nonopioid pain management benefits.^6,8^ The SSBCIs were transitional or temporary supports, food and produce benefits, meals beyond a limited basis, pest control, transportation for nonmedical needs, indoor air quality, social needs, complementary therapies, services supporting self-direction, structural home modifications, or general supports for living.^9,12^ The outcome of interest was an enrollee’s plan rating. The MA CAHPS survey includes an item for which respondents rate their health plan on a 0- to 10-point response scale, with 0 indicating the worst health plan possible and 10 indicating the best health plan possible.

We controlled for individual-, plan-, and county-level variables associated with plan ratings.^14,15,16,17,18,19,20^ We included the MA CAHPS case-mix adjustment variables that addressed differences in the enrollee populations across plans: age (<65, 65-74, 75-84, and ≥85 years), educational attainment (eighth grade or less, some high school, high school graduation or General Educational Development, some college or a 2-year degree, 4-year college, or more than a 4-year degree), physical and mental health (poor, fair, good, very good, or excellent), and receipt of proxy assistance (eg, whether a proxy helped an enrollee complete the survey or answer questions).^21^ We also included dual eligibility and self-reported sex, race and ethnicity (American Indian or Alaska Native, Asian, Hispanic, Native Hawaiian or Other Pacific Islander; non-Hispanic Black, non-Hispanic White, or unknown), hospitalization, living status, and diagnosed health conditions (myocardial infarction, high blood pressure, diabetes, chronic obstructive pulmonary disorder, cancer, or angina). We opted to account for race and ethnicity in our sample given the increasing enrollment patterns of Black and Hispanic Medicare beneficiaries into the MA program. We accounted for the number of days of plan enrollment before survey completion.

We accounted for differences in plan structure associated with member experience. We included plan-level indicators for SNP status and plan type as binary variables and county-level enrollment, premium, maximum OOP spending, deductible, and changes in premium, deductible, and maximum OOP spending from the previous year as continuous variables.^17,21,22^ We adjusted for plan size defined by 3 groups of plan enrollment. We adjusted for contract start year as before 2006, 2006 to 2013, or 2014 to 2019, based on significant changes to the MA program, including the Medicare Modernization Act in 2006 and the MA Quality Improvement Program under the Patient Protection and Affordable Care Act in 2013. Finally, we adjusted for MA penetration to account for differences in the availability and popularity of MA plans across counties.

### Statistical Analysis

We compared baseline characteristics of enrollees in plans that adopted a PHRB, SSBCI, or both benefits vs enrollees in plans that did not adopt a supplemental benefit. Next, we analyzed changes in enrollees’ plan ratings using linear probability models with difference-in-differences estimators. To be valid, the preperiod trends needed to be parallel. We tested this assumption graphically and statistically and found no significant difference in preperiod trends between treatment and control plans (Figure; eTable 2 in Supplement 1). The unit of analysis was the MA CAHPS survey respondent. We included county, year, and plan fixed effects to account for differences within counties and plans and across time. We adjusted SEs for plan-level clustering. We fit separate models for each benefit.

**Figure.**
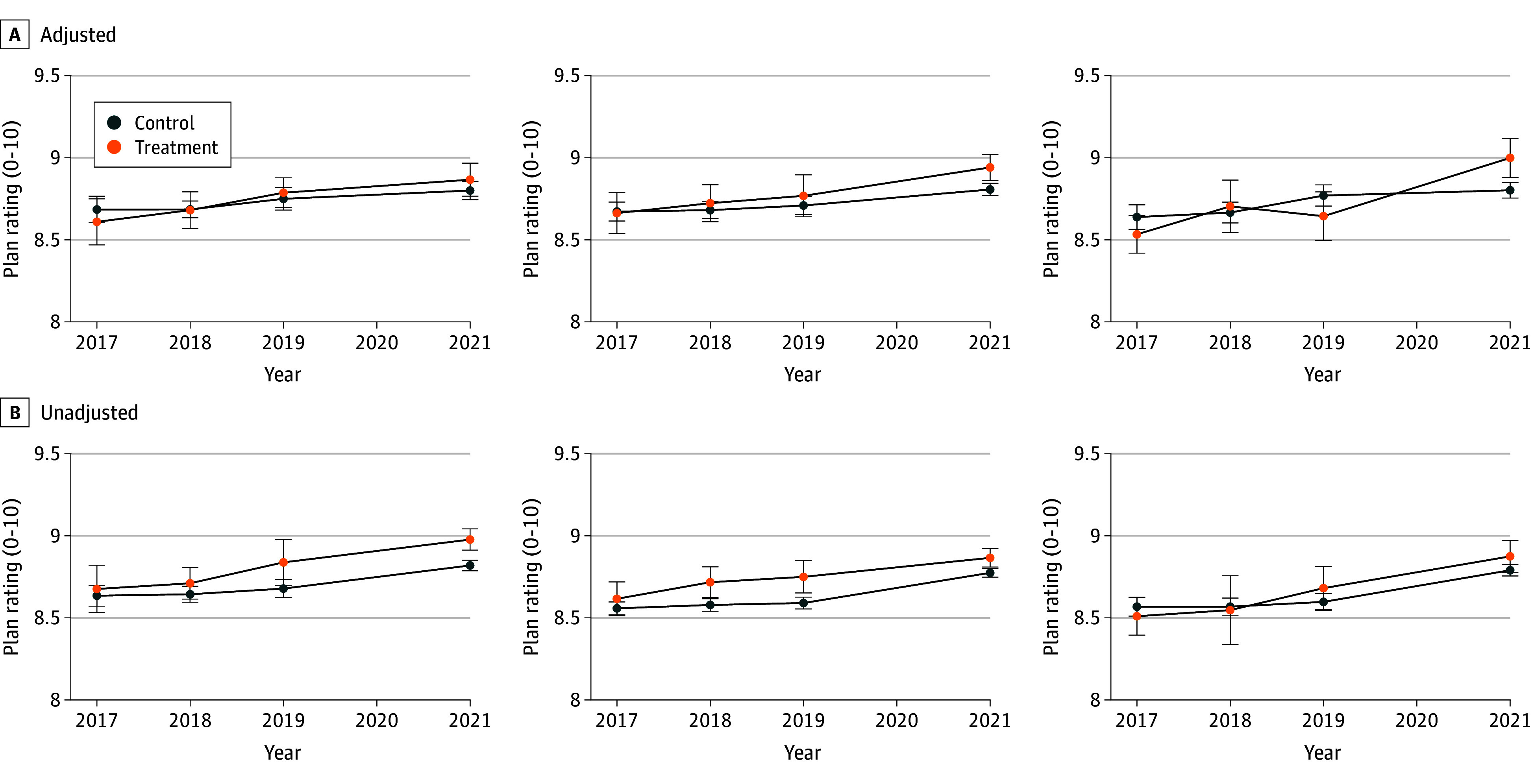
Parallel Trends Validation of the Primarily Health-Related Benefit, Special Supplemental Benefit for the Chronically Ill, and Both Benefits Cohorts, 2017-2021 The figure shows adjusted (A) and unadjusted (B) plan ratings on a scale of 1 to 10, with 0 indicating the worst health plan possible and 10 indicating the best health plan possible. Data are from the authors’ analysis of 2017-2021 Medicare Advantage Consumer Assessments of Healthcare Providers and Systems survey data and Medicare administrative enrollment data and publicly available Medicare Advantage plan benefit, enrollment, and crosswalk data. Trends reflect results from the fully adjusted models accounting for individual-level (sex, race and ethnicity, age, original reason for entitlement, dual eligibility, educational attainment, self-rated physical and mental health, receipt of proxy assistance, self-reported hospitalization utilization, living alone, self-reported chronic conditions, US Census region, inpatient admissions, nursing home admissions, home health visits, chronic conditions, and number of days in 2019 an enrollee was in the plan) and plan-level (plan type, plan size, Special Needs Plan status, contract start year, change in maximum out-of-pocket spending, change in premium, change in deductible, and plan age) factor. We tested this assumption statistically with the Wald 2-tailed, unpaired *t* test, testing the null hypothesis that the slopes in the plans that did and did not adopt supplemental benefits did not change differentially across the preperiod. This test was not statistically significant (*P* < .05) across outcomes for primarily health-related benefits (2018: *P* = .93; 2019: *P* = .89), Special Supplemental Benefits for the Chronically Ill (2018: *P* = .31; 2019: *P* = .09), and both benefits (2018: *P* = .19; 2019: *P* = .41). Error bars indicate 95% CIs.

We conducted 6 sensitivity analyses. First, we modeled nonresponse to the plan-rating item in our linear probability model to test its association with supplemental benefit adoption. Second, we analyzed the association between PHRB adoption in 2019, which was the first year of potential adoption, and plan ratings. Third, we analyzed plans that adopted a PHRB, SSBCI, or both benefits throughout the study period (2019-2021 for PHRB, 2020-2021 for SSBCI, or both). Fourth, we assessed whether there was a dose-response association between plans that adopted multiple benefits and member experience analyzing the association between plans adopting 2 or more PHRBs or SSBCIs or 2 or more of both in 2021 that had not adopted any benefits prior. Fifth, we assessed the association between adoption of a PHRB, SSBCI, and both in 2021 and members’ self-reported health and health care utilization. Sixth, we included an event study analysis to account for whether a plan adopted a PHRB in 2019 to 2021, an SSBCI in 2020 to 2021, or both in 2020 to 2021. All analyses accounted for the probability of selection, variations in subgroup representation, and disproportionate responses among specific subgroups (eg, non-Hispanic White individuals, older individuals, and low-income enrollees) using plan-level survey weights.^23,24,25,26,27^ Analyses were performed between April 2023 and March 2024 in Stata version 18.0 (StataCorp LLC). All tests were 2-sided with a *P* < .05 significance level.

## Results

There were 388 356 responses across waves (Table 1). These responses represented individuals from 467 contracts and 2558 plans in 2021 (eTable 3 in Supplement 1). Among respondents, the mean (SD) age was 74.6 (8.7) years; 57.2% were female; 8.9% were fully Medicare-Medicaid dual eligible and 6.2% were partially dual eligible; 74.6% self-reported at least 1 chronic condition; 13.7% had less than a high school diploma; 9.7% had help from a proxy; 45.0% had fair or poor self-rated physical health; 58.1% had fair or poor self-rated mental health; 15.6% were entitled to Medicare originally due to disability; 7.8% were younger than 65 years; 4.2% were Asian; 10.5% were Black; 12.5% were Hispanic, 2.3% were American Indian, Alaska Native, Native Hawaiian, or Other Pacific Islander; 70.4% were White; 0.2% were of unknown race; 31.8% lived alone; and 11.0% were enrolled in an SNP (eTable 3 in Supplement 1). There were differences across cohorts as shown in the stratified sample characteristics (Table 1). For the PHRB cohort, enrollees in the treatment group were more likely to be Hispanic, older than 85 years, partially or fully dual eligible, helped by a proxy, and living alone (Table 1). For the SSBCI and both benefit cohorts, enrollees in the treatment groups were more likely to be of races other than White, to be younger than 65 years, to be eligible for Medicare due to disability, to be partially dual eligible, to have received less than a high school diploma, to have received help from a proxy to complete the survey, to live alone, and to be enrolled in an SNP (Table 1).

**Table 1.** Sample Characteristics for Enrollees in Treatment and Control Plans^a^

Characteristic	PHRB, 2021		SSBCI, 2021		Both benefits, 2021	
	Adopted plan	Did not adopt plan	Adopted plan	Did not adopt plan	Adopted plan	Did not adopt plan
Survey weighted sample size, No.	9393	71 388	16 888	164 963	7450	78 843
Sample size, No.	16 862	63 918	30 936	150 916	10 711	76 802
Plans, No.	80	1012	115	1603	91	1082
**Individual characteristics**						
Sex						
Female	57.8	56.5	58.8^b^	56.5	56.7	56.5
Male	42.2	43.5	41.2^b^	43.5	43.3	43.5
Race and ethnicity						
Asian	1.1^b^	3.5^b^	2.5^b^	3.5^b^	9.5^b^	4.3^b^
Black	6.4^b^	8.5^b^	17.4^b^	8.5^b^	8.6^c^	8.9^c^
Hispanic	16.3^b^	11.4^b^	22.1^b^	11.4^b^	27.2^b^	14.9^b^
American Indian, Alaska Native, Native Hawaiian, or Other Pacific Islander	1.8^b^	2.6^b^	2.4^b^	2.6^b^	2.4^d^	2.4^d^
White	74.2	73.8	55.4	73.8	52.2	70.0
Unknown	0.2	0.2	0.2	0.2	0.1	0.1
Age group, y						
<65	7.6	7.6	12.0^b^	7.6	14.9^b^	7.4
65-74	44.7	47.5	42.3^b^	47.5	43.7^b^	48.0
75-84	33.0^d^	31.7^d^	32.8^b^	31.7	29.0^b^	31.6
≥85	14.7^c^	13.2^c^	12.9^b^	13.2	12.4^b^	13.0
Entitlement						
Old age	86.3	85.8	78.8	85.7	75.2	86.1
Disability	13.5	14.0	21.2^c^	14.1^c^	24.6^b^	14.9^b^
ESRD	0.1	0.1	0.0^d^	0.1^d^	0.1	0.1
Disability and ESRD	0.1	0.0	0.1	0.1	0.1	0.0
Dual eligibility						
Partial dual	8.1^b^	5.9^b^	15.0^b^	4.9^b^	14.0^b^	4.5^b^
Full dual	10.8^b^	4.9^b^	19.7^b^	5.9^b^	7.3	7.3
Educational level						
Less than high school diploma	15.4^b^	12.7^b^	26.6^b^	12.0^b^	23.7^b^	12.4^b^
High school diploma	36.5^b^	27.3^b^	33.2^b^	31.2^b^	30.7^b^	27.4^b^
More than high school diploma	48.1^b^	60.0^b^	40.2^b^	56.8^b^	45.6^b^	60.2^b^
Chronic conditions						
Myocardial infarction	8.2	8.6	8.5^d^	8.6^d^	8.5	8.1
High blood pressure	59.5	59.0	61.6^b^	59.0^b^	58.3	57.8
Diabetes	25.6^b^	28.2^b^	30.1^b^	28.2^b^	31.9^b^	27.6^b^
COPD	15.7	16.1	17.0^b^	16.1^b^	17.2^c^	14.7^c^
Cancer	12.4	13.1	11.3^b^	12.7^b^	9.0^b^	12.7^b^
Angina	13.2	13.2	13.7^c^	12.4^c^	12.6	12.2
Mental health						
Poor	25.1	27.3	21.2	27.3	22.0	26.9
Fair	32.4	32.3	28.2	32.2	27.1^b^	33.2^b^
Good	28.0^d^	27.2^d^	30.7^b^	27.3^b^	29.9^b^	27.0^b^
Very good	12.6^c^	11.0^c^	16.7^b^	11.0^b^	17.2^b^	11.0^b^
Excellent	1.9	2.2	3.2^b^	2.2^b^	3.8^b^	1.9^b^
Physical health						
Poor	8.1	8.1	6.9	8.2	7.4	8.9
Fair	27.6	28.9	22.3	28.9	22.3	29.2
Good	39.1	38.6	37.3^b^	38.6^b^	34.9	38.0
Very good	20.7	20.0	27.7^b^	19.9^b^	28.8^b^	20.1^b^
Excellent	4.5	4.4	5.8^b^	4.4^b^	6.6^b^	4.1^b^
Proxy status						
Proxy helped	9.4^d^	8.4^d^	14.4^b^	8.4^b^	14.8^b^	9.0^b^
Proxy answered	3.4	3.1	4.1^c^	3.1^c^	4.4^c^	3.1^c^
Hospital utilization						
None	87.3^d^	88.5^d^	87.2^d^	88.5^d^	87.8	88.5
≥1 Visit	12.7^d^	11.5^d^	12.8^d^	11.5^d^	12.2	11.5
Lives alone	33.4^c^	30.5^c^	36.1^b^	30.5^b^	32.0^c^	29.0^c^
Time in plan before survey, d	104^b^	102^b^	102	102	105^b^	102^b^
**Plan characteristics**						
Plan type						
HMO	72.3^b^	58.3^b^	80.9^b^	58.3^b^	91.1^b^	66.6^b^
PPO	27.7^b^	41.7^b^	19.1^b^	41.7^b^	8.9^b^	33.4^b^
Plan size						
Small (<750)	1.4	1.4	1.4	1.4	2.5	1.1
Medium (750-4300)	16.3^b^	11.5^b^	10.4	11.5	13.4^d^	9.0^d^
Large (>4300)	82.3	87.1	88.2^d^	87.1^d^	84.2^b^	89.9^b^
SNP	6.3^b^	9.7^b^	26.6^b^	9.7^b^	29.7^b^	8.6^b^
Contract start						
Before 2006	78.5^b^	61.8^b^	63.7^b^	61.8^b^	55.7^b^	61.1^b^
2006-2013	12.9^b^	35. ^b^	30.4^b^	35.8^b^	43.6^b^	35.9^b^
2014-2020	8.6^b^	2.4^b^	5.9^b^	2.4^b^	0.7^b^	3.0^b^
Maximum OOP spending, mean (SD), $	4881 (1339)^b^	5692 (1473)^b^	5455 (1733)^b^	5560 (1502)^b^	4360 (1635)^b^	5286 (1638)^b^
Change in maximum OOP spending, mean (SD), $^e^	6 (585)^b^	−107 (630)^b^	−13 (344)	−10 (541)	−432 (1077)^b^	−93 (601)^b^
Premium, mean (SD), $	25 (51)^b^	6 (21)^b^	20 (38)^b^	10 (29)^b^	4 (15)^b^	5 (18)^b^
Change in premium, mean (SD), $^e^	0.2 (7)	−4 (15)	0 (10)	−3 (16)	−0.3 (5)	−3 (15)
Deductible, mean (SD), $	1 (24)^b^	54 (223)^b^	89 (255)^b^	33 (164)^b^	7 (32)^b^	49 (219)^b^
Change in deductible, mean (SD, $^e^	−8 (34)^c^	−2 (71)^c^	−11 (83)	−11 (132)	−0.1 (3)^b^	−3 (80)^b^
**County characteristics**						
County-level enrollment, mean (SD), No.	16 430 (11 555)^b^	98 388 (153 244)^b^	20 718 (15 143)^b^	103 979 (158 673)^b^	15 871 (11 878)^b^	111 369 (158 562)^b^
MA penetration	51.7^b^	48.1^b^	43.3^d^	43.7^d^	49.6^b^	46.8^b^

Abbreviations: COPD, chronic obstructive pulmonary disorder; ESRD, end-stage renal disease; HMO, health maintenance organization; MA, Medicare Advantage; OOP, out of pocket; PHRB, primarily health-related benefit; PPO, preferred provider organization; SNP, Special Needs Plan; SSBCI, Special Supplemental Benefit for the Chronically Ill.

^a^ Data are from the authors’ analysis of 2017 to 2021 MA Consumer Assessment of Health Care Providers and Systems survey data and Medicare administrative enrollment data and publicly available MA plan benefit, enrollment, and crosswalk data using plan-level MA Consumer Assessment of Health Care Providers and Systems survey weights. Individual-level data are based on enrollee self-report. Data are presented as percentages unless otherwise indicated.

^b^ *P* < .001.

^c^ *P* < .01.

^d^ *P* < .05.

^e^ Change reflects the difference between the base year and the year prior for max OOP, premium, and deductible.

Across cohorts, plan rating increased over time (Table 2). Enrollees in plans that adopted a PHRB had a mean (SD) plan rating of 8.81 (1.54) points out of 10 at baseline and controls had a mean (SD) rating of 8.69 (1.64) points (Table 2). After 2021, enrollees in plans that adopted a PHRB had a mean (SD) plan rating of 8.98 (1.44) points, and controls had a mean (SD) rating of 8.79 (1.60) points (Table 2). Enrollees in plans that adopted an SSBCI had a mean (SD) plan rating of 8.74 (1.70) points at baseline, and controls had a mean (SD) rating of 8.68 (1.66) points (Table 2). After 2021, enrollees in plans that adopted an SSBCI had a mean (SD) plan rating of 8.98 (1.56) points, and controls had a mean (SD) rating of 8.81 (1.55) points (Table 2). There was no association between plans that adopted a PHRB (adjusted difference, −0.12 points; 95% CI, −0.26 to 0.02 points) or SSBCI (adjusted difference, 0.09 points; 95% CI, −0.03 to 0.21 points) and a change in mean enrollee plan rating (Table 2). Enrollees in plans that adopted both benefits had a mean (SD) plan rating of 8.70 (1.75) at baseline, and controls had a mean (SD) rating of 8.70 (1.65) points (Table 2). After 2021, enrollees in plans that adopted both benefits had a mean (SD) plan rating of 8.97 (1.46) points, and controls had a mean (SD) rating of 8.80 (1.59) (Table 2). Among plans that adopted both benefits, in adjusted analyses, the mean enrollee plan rating increased 0.22 of 10 points (95% CI, 0.04-0.40 points) (Table 2).

**Table 2.** Unadjusted and Adjusted Impact of PHRB, SSBCI, or Both Benefit Adoption on Plan Rating, 2021^a^

Benefit	Treatment		Control		Unadjusted DiD	Adjusted DID (95% CI)^b^
	Preperiod (2017-2019)	Postperiod (2021)	Preperiod (2017-2019)	Postperiod (2021)		
**PHRB**						
Plan rating, mean (SD)	8.81 (1.54)	8.98 (1.44)	8.69 (1.64)	8.79 (1.60)	0.07	−0.12 (−0.26 to 0.020)
Sample, No.^c^	16 892	9900	63 918	50 767	NA	NA
**SSBCI**						
Plan rating, mean (SD)	8.74 (1.66)	8.98 (1.56)	8.68 (1.66)	8.81 (1.55)	0.11	0.09 (−0.03 to 0.210)
Sample, No.^c^	30 936	11 191	150 915	79 856	NA	NA
**Both benefits**						
Plan rating, mean (SD)	8.70 (1.75)	8.97 (1.46)	8.70 (1.65)	8.80 (1.59)	0.17	0.22 (0.04 to 0.40)^d^
Sample, No.^c^	10 711	4608	76 083	48 072	NA	NA

Abbreviations: DID, difference in differences; NA, not applicable; PHRB, primarily health-related benefit; SSBCI, Special Supplemental Benefit for the Chronically Ill.

^a^ Data are from the authors’ analysis of 2017 to 2021 Medicare survey data and administrative enrollment data and publicly available Medicare Advantage plan benefit, enrollment, and crosswalk data. This table presents the unadjusted and adjusted DID estimates of the association of PHRB, SSBCI, or both benefits adoption with individual plan rating expressed as the mean plan rating across treatment and control plans.

^b^ Fully adjusted models control for individual-level (sex, race and ethnicity, age, original reason for entitlement, dual eligibility, educational attainment, self-rated physical and mental health, receipt of proxy assistance, self-reported hospitalization utilization, living alone, self-reported chronic conditions, US Census region, inpatient admissions, nursing home admissions, home health visits, chronic conditions, and number of days in 2021 an enrollee was in the plan), plan-level factors (plan type, plan size, Special Needs Plan status, contract start year, change in maximum out-of-pocket spending, change in premium, change in deductible, and plan age), and county Medicare Advantage penetration rate.

^c^ The number of person-years in each treatment group.

^d^ *P* < .05.

The results of the sensitivity analyses are available in eTables 4-10 in Supplement 1. In adjusted analyses, nonresponse was not associated with supplemental benefit adoption across benefits (eTable 4 in Supplement 1). The 2017-2019 PHRB analysis findings were nonsignificant and similar in direction with a smaller magnitude to the main analysis (eTable 5 in Supplement 1). When accounting for any benefit adoption between 2019 and 2021, the results were similar in direction, with a smaller magnitude, and there was no statistically significant association between enrollee plan rating and a plan adopting both benefits (eTable 6 in Supplement 1). In fully adjusted models, there was no dose-response association between benefit adoption and plan rating; however, in unadjusted models, plans that adopted 2 or more PHRBs and SSBCIs in 2021 that had not previously adopted any saw an increase of 0.53 out of 10 points (95% CI, 0.08-0.97 points) in mean plan ratings (eTable 7 in Supplement 1). There was no statistically significant association between benefit adoption and health care utilization or self-rated health across metrics (eTable 8 in Supplement 1). In the event study analysis, there was no statistically significant association between benefit adoption and member experience in the preperiod or postperiod (eFigures 2-7 in Supplement 1). Individuals in plans that adopted a supplemental benefit were slightly more likely to report that their plan offered them a benefit for their health condition in the treatment year across benefit types (eTables 9 and 10 in Supplement 1).

## Discussion

We found that plans that adopted both a PHRB and SSBCI benefit saw an increase in the mean enrollee plan rating of 0.22 point of 10. However, we found no statistically significant association between plans adopting only a PHRB or SSBCI and a change in mean enrollee plan ratings. Similar to prior research using CAHPS data to evaluate patient experience, we applied the heuristic of 0.1 point to indicate a small, 0.3 points a medium, and 0.5 points a large difference in quality.^21,28^ We found that the differences attributable to plans adopting both benefits were small to medium.

Plans that adopted both benefits saw a small to medium 0.22-point increase in plan ratings. It is possible that plans adopting both types of benefits were more invested in the potential of the supplemental benefits to support their members, which may have translated to activities, including marketing and outreach, that increased member awareness and uptake. The supplemental benefits are designed to address social risks and improve the health of socially complex beneficiaries. In qualitative interviews, plan leadership described the ability to increase membership, retain members, and meet consumer expectations as drivers of potential investments in SSBCIs.^29^ Because high-needs enrollees disproportionately disenroll from MA, the expanded benefits may help plans to retain those members. In competitive MA markets, the benefits may help to attract people who are more medically or socially complex.^30,31,32^ Another explanation is that enrollees who selected these plans did so in part because of the supplemental benefits, leading their plan ratings to be more closely tied to the benefits.

Plans that adopted either a PHRB or SSBCI saw no statistically significant change in the mean enrollee plan rating. This finding could be explained by a lack of enrollee awareness: using data from the MA CAHPS, we found marginal differences between plans that did and did not adopt a PHRB or SSBCI and whether an enrollee reported receiving an extra benefit from their health plan to manage a health condition. It could also be explained by cautiousness by the plans. Plans in our sample were later adopters; they adopted a PHRB or SSBCI for the first time in 2021 (2 years after PHRBs and 1 year after SSBCIs were allowed). Plans may have been cautious to invest in and market the new benefits without insights into how they impact member experience and plan selection. Furthermore, MA plans are financially incentivized to recruit and retain less costly members, which may have led them to invest more in benefits that ensured lower-cost enrollees had positive experiences rather than investments ensuring positive experiences for more complex enrollees. Historically, MA plans adopted fitness memberships to attract “good risk” (ie, healthier members).^33^ Plans that adopted only 1 type of benefit may not have sufficiently focused on the new supplemental benefits to change the experiences of the enrollees who would most benefit from them.^34,35,36^

Responses from the MA CAHPS survey previously comprised a third of the measures in the MA star rating calculations.^22^ Since 2020, the CMS gradually increased the weight of survey responses reaching the target weighting in 2023.^37^ As plans face the loss of pandemic-era flexibilities in star rating calculations, they may look for mechanisms to increase plan experience ratings and other metrics that drive star ratings and bonus payments.^38^

Our findings add to the growing body of literature on factors associated with MA enrollee experience. To facilitate further research, the CMS could provide access to data on the adoption, scope, availability, and uptake of the benefits. When these data are available, studies should explore the impact of the supplemental benefits on enrollees’ health care experiences and utilization and other factors associated with changes in enrollees’ plan experiences.

## Limitations

This study has several limitations. First, we evaluated the role of plan-level adoption of a supplemental benefit on plan rating, but we cannot assess who in the plan selected the plan based on or used the benefits. Because our study design isolates the association of adopting these benefits from other plan characteristics, we provided initial insights into the association between supplemental benefit adoption and plan experience. Second, we could not include data from 2020 because the MA CAHPS survey was not performed due to the global COVID-19 pandemic. We did not expect that the virus had differential associations on enrollees in our treatment and control groups. Third, because the survey typically runs between March and June, our data may provide a lower bound of the association between supplemental benefit adoption with plan rating. To account for this variation, we controlled for each enrollee’s number of enrolled days before being surveyed annually. Fourth, we used survey data, which are vulnerable to nonresponse bias and subgroup variations in responses. After survey weights were applied, there were modest differences between responders and nonresponders.^18,19,25^ We found that supplemental benefit adoption was not associated with changes in nonresponse or differentially with the treatment and control groups. Fifth, we controlled for changes in plan offerings (eg, maximum OOP spending, deductible, or premium) but may not have fully isolated the effects of supplemental benefit adoption from other changes plans made.

## Conclusions

In this cohort study, we found that plans that implemented both supplemental benefits saw a small to medium increase in their overall plan rating compared with plans that did not. This evidence suggests that more investments in supplemental benefits were associated with improved plan experiences, which could contribute to improved plan quality ratings.

## Notes

Supplement 1. **eFigure 1.** Sample Inclusion and Exclusion Criteria **eTable 1.** Contract-Level, Plan-Level, and Enrollment-Level Adoption of PHRB and SSBCI Benefits **eTable 2.** Difference-in-Differences Parallel Trends Validation, 2021 **eTable 3.** Sample Characteristics for Enrollees in Treatment and Control Plans at Baseline, 2017-2019 **eTable 4.** Unadjusted and Adjusted Impact of PHRB, SSBCI, or Both Benefit Adoption on Probability of Non-Response in the MA CAHPS, 2017-2021 **eTable 5.** Unadjusted and Adjusted Impact of PHRB Adoption on Plan Rating, 2019 **eTable 6.** Unadjusted and Adjusted Impact of PHRB, SSBCI, or Both Benefit Adoption on Plan Rating, 2017-2021 **eTable 7.** Unadjusted and Adjusted Impact of PHRB, SSBCI, or Both Benefit Adoption on Plan Rating for Plans that Adopted Two or More Benefits, 2021 **eTable 8.** Unadjusted and Adjusted Impact of PHRB, SSBCI, or Both Benefit Adoption on Health Utilization and Self-Reported Health, 2021 **eFigure 2.** Adjusted Event Study for Primarily Health Related Benefit (PHRB) Adoption, 2019-2021 **eFigure 3.** Unadjusted Event Study for Primarily Health Related Benefit (PHRB) Adoption, 2019-2021 **eFigure 4.** Adjusted Event Study for Special Supplemental Benefits for the Chronically Ill (SSBCI) Adoption, 2020-2021 **eFigure 5.** Unadjusted Event Study for Special Supplemental Benefits for the Chronically Ill (SSBCI) Adoption, 2020-2021 **eFigure 6.** Adjusted Event Study for Plans that Adopted Both Benefits, 2020-2021 **eFigure 7.** Unadjusted Event Study for Plans that Adopted Both Benefits, 2020-2021 **eTable 9.** Rate of Extra Benefit Availability Due to Health Condition Across Benefit Cohort, 2017-2021 **eTable 10.** Relationship Between Supplemental Benefit Adoption and Enrollee Receipt of Extra Benefit Due to Health Condition, 2017-2021

Supplement 2.
Data Sharing Statement
