# Supplementary material for: New Supplemental Benefits and Plan Ratings Among Medicare Advantage Enrollees
Source: JAMA Netw Open. 2024 Jun 5;7(6):e2415058. doi: 10.1001/jamanetworkopen.2024.15058 (PMC11154155; doi:10.1001/jamanetworkopen.2024.15058)
Supplement: Supplement 1. — eFigure 1. Sample Inclusion and Exclusion Criteria eTable 1. Contract-Level, Plan-Level, and Enrollment-Level Adoption of PHRB and SSBCI Benefits eTable 2. Difference-in-Differences Parallel Trends Validation, 2021 eTable 3. Sample Characteristics for Enrollees in Treatment and Control Plans at Baseline, 2017-2019 eTable 4. Unadjusted and Adjusted Impact of PHRB, SSBCI, or Both Benefit Adoption on Probability of Non-Response in the MA CAHPS, 2017-2021 eTable 5. Unadjusted and Adjusted Impact of PHRB Adoption on Plan Rating, 2019 eTable 6. Unadjusted and Adjusted Impact of PHRB, SSBCI, or Both Benefit Adoption on Plan Rating, 2017-2021 eTable 7. Unadjusted and Adjusted Impact of PHRB, SSBCI, or Both Benefit Adoption on Plan Rating for Plans that Adopted Two or More Benefits, 2021 eTable 8. Unadjusted and Adjusted Impact of PHRB, SSBCI, or Both Benefit Adoption on Health Utilization and Self-Reported Health, 2021 eFigure 2. Adjusted Event Study for Primarily Health Related Benefit (PHRB) Adoption, 2019-2021 eFigure 3. Unadjusted Event Study for Primarily Health Related Benefit (PHRB) Adoption, 2019-2021 eFigure 4. Adjusted Event Study for Special Supplemental Benefits for the Chronically Ill (SSBCI) Adoption, 2020-2021 eFigure 5. Unadjusted Event Study for Special Supplemental Benefits for the Chronically Ill (SSBCI) Adoption, 2020-2021 eFigure 6. Adjusted Event Study for Plans that Adopted Both Benefits, 2020-2021 eFigure 7. Unadjusted Event Study for Plans that Adopted Both Benefits, 2020-2021 eTable 9. Rate of Extra Benefit Availability Due to Health Condition Across Benefit Cohort, 2017-2021 eTable 10. Relationship Between Supplemental Benefit Adoption and Enrollee Receipt of Extra Benefit Due to Health Condition, 2017-2021 [file jamanetwopen-e2415058-s001.pdf]

Supplementary Online Content

Tucher EL, Meyers DJ, Trivedi AN, Gottlieb LM, Thomas KS. New supplemental benefits and plan ratings among Medicare Advantage enrollees. *JAMA Netw Open*. 2024;7(6):e2415058. doi:10.1001/jamanetworkopen.2024.15058

eFigure 1. Sample Inclusion and Exclusion Criteria
eTable 1. Contract-Level, Plan-Level, and Enrollment-Level Adoption of PHRB and SSBCI Benefits
eTable 2. Difference-in-Differences Parallel Trends Validation, 2021
eTable 3. Sample Characteristics for Enrollees in Treatment and Control Plans at Baseline, 2017-2019
eTable 4. Unadjusted and Adjusted Impact of PHRB, SSBCI, or Both Benefit Adoption on Probability of Non-Response in the MA CAHPS, 2017-2021
eTable 5. Unadjusted and Adjusted Impact of PHRB Adoption on Plan Rating, 2019
eTable 6. Unadjusted and Adjusted Impact of PHRB, SSBCI, or Both Benefit Adoption on Plan Rating, 2017-2021
eTable 7. Unadjusted and Adjusted Impact of PHRB, SSBCI, or Both Benefit Adoption on Plan Rating for Plans that Adopted Two or More Benefits, 2021
eTable 8. Unadjusted and Adjusted Impact of PHRB, SSBCI, or Both Benefit Adoption on Health Utilization and Self-Reported Health, 2021
eFigure 2. Adjusted Event Study for Primarily Health Related Benefit (PHRB) Adoption, 2019-2021
eFigure 3. Unadjusted Event Study for Primarily Health Related Benefit (PHRB) Adoption, 2019-2021
eFigure 4. Adjusted Event Study for Special Supplemental Benefits for the Chronically Ill (SSBCI) Adoption, 2020-2021
eFigure 5. Unadjusted Event Study for Special Supplemental Benefits for the Chronically Ill (SSBCI) Adoption, 2020-2021
eFigure 6. Adjusted Event Study for Plans that Adopted Both Benefits, 2020-2021
eFigure 7. Unadjusted Event Study for Plans that Adopted Both Benefits, 2020-2021
eTable 9. Rate of Extra Benefit Availability Due to Health Condition Across Benefit Cohort, 2017-2021
eTable 10. Relationship Between Supplemental Benefit Adoption and Enrollee Receipt of Extra Benefit Due to Health Condition, 2017-2021

This supplementary material has been provided by the authors to give readers additional information about their work.

© 2024 Tucher EL et al. *JAMA Network Open.*

eFigure 1. Sample Inclusion and Exclusion Criteria

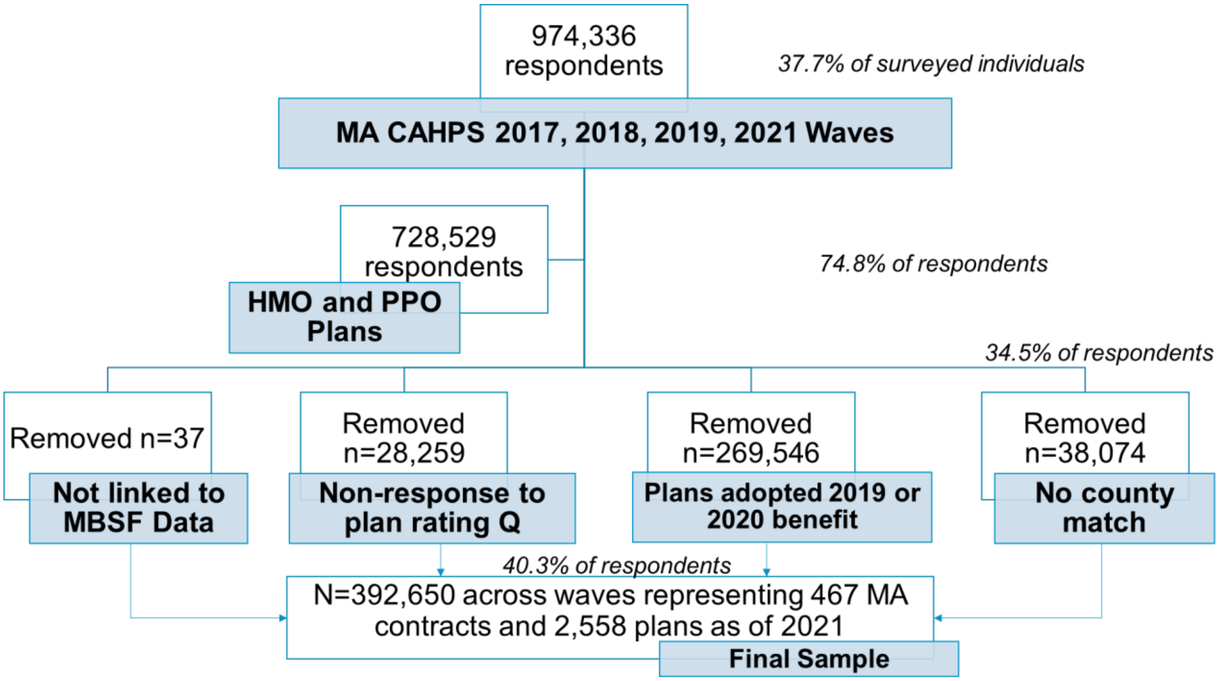

SOURCE Authors' analysis of 2017-2021 Medicare Advantage (MA) Consumer Assessment of Health Care Providers and Systems (CAHPS) survey data and Medicare administrative enrollment data and publicly available MA plan benefit, enrollment, and crosswalk data. NOTES: HMO: Health Maintenance Organization; PPO: Preferred Provider Organization; MBSF: Medicare Beneficiary Summary File. We excluded individuals enrolled in Medicare-Medicaid, PACE, MSA, PFFS, PDP, Cost, and employer-sponsored plans (n=245,807).

© 2024 Tucher EL et al. *JAMA Network Open.*

eTable 1. Contract-Level, Plan-Level, and Enrollment-Level Adoption of PHRB and SSBCI Benefits

Benefit	Contracts (n=348)	Plans (n=1,484)	Enrollees (n=60,667)
**PHRB, %**			
In-home support	9.2	5.4	9.4
Home-based palliative care	2.9	2.4	7.3
Non-opioid pain management	4.9	2.0	2.1
Caregiver support	1.4	1.0	1.1
Adult day care	0.0	0.0	0.0
Any new benefit	16.4	9.5	16.3
**Benefit**	**Contracts (n=413)**	**Plans (n=1,992)**	**Enrollees (n=91,047)**
**SSBCI, %**			
Produce	8.7	3.0	5.4
Meals	5.1	5.0	5.0
Non-medical transportation	2.7	0.9	1.7
Social need	4.6	1.3	1.7
Pest control	0.7	0.3	0.4
Temporary Supports	1.0	0.4	0.3
Air quality control	0.5	0.2	0.1
Service dog support	0.0	0.0	0.0
Self-directed services	0.2	0.1	0.0
Complimentary Therapies	0.0	0.0	0.0
Home modifications	0.0	0.0	0.0
Any new benefit	15.7	8.8	12.3

SOURCE Authors' analysis of 2017-2021 MA CAHPS survey data and Medicare administrative enrollment data and publicly available MA plan benefit, enrollment, and crosswalk data.

eTable 2. Difference-in-Differences Parallel Trends Validation, 2021

	Unadjusted	Adjusted	p-value	95% CI
**Expanded Primarily Health Related Benefits (PHRBs)**				
2018#Treat	0.04	0.01	0.93	-0.15, 0.17
2019#Treat	0.12	0.01	0.89	-0.15, 0.17
**Special Supplemental Benefits for the Chronically Ill (SSBCIs)**				
2018#Treat	0.06	0.03	0.31	-0.03, 0.10
2019#Treat	0.07	0.09	0.09	-0.01, 0.19
**Adopted Both Benefits (Both)**				
2018#Treat	0.11	0.10	0.23	-0.06, 0.25
2019#Treat	-0.11	0.06	0.48	-0.10, 0.22

SOURCE Authors' analysis of 2017-2021 MA CAHPS survey data and Medicare administrative enrollment data and publicly available MA plan benefit, enrollment, and crosswalk data. NOTES: The p-values are from linear probability models estimating the interaction of treatment group and year in the pre-period to test if the parallel trends assumption was met. P>0.05 shows there does not appear to be significant differences in parallel trends. CI: confidence interval.

eTable 3. Sample Characteristics for Enrollees in Treatment and Control Plans at Baseline, 2017-2019

Variable	Treatment		Control
Survey Weighted Sample Size, N (%)	37,765 (14.3)		220,589 (85.7)
Sample, N (%)	58,509 (22.7)		198,846 (77.3)
	**Individual Characteristics**		
Female, %		*	
	58.0		56.8
Race, %			
White	59.4***		72.2***
Black	12.2***		10.0***
Hispanic	21.9***		11.2***
Asian	4.0***		3.9***
Native American/ Pacific Islander	2.3***		2.5***
Other	0.2		0.2
Age, %		***	
Under 65	11.6		7.4
65-74	43.3		47.8
75-84	31.9		31.6
Over 85	13.2		13.2
Entitlement, %			
Old Age	79.8		85.9
Disability	20.0***		14.0***
ESRD	0.1		0.0
Disability and ESRD	0.1		0.1
Dual Eligibility, %		***	
Partial Dual	13.0		8.0
Full Dual	14.1		4.8
Education		***	
<High School Diploma	22.9		12.9
High School Diploma	33.4		29.4
>High School Diploma	43.7		57.7
Chronic Conditions, %			
Heart Attack	8.4		8.2
High Blood Pressure	60.2*		59.0*
Diabetes	29.4***		27.6***
COPD	16.7***		14.8***
Cancer	11.0		12.7
Angina	13.2		12.8
Self-Rated Mental Health			
Poor	22.4		26.7
Fair	29.0		33.2
Good	29.8***		27.0***
Very Good	15.8***		11.0***
Excellent	3.0***		2.1***
Self-Rated Physical Health			
Poor	7.3		8.5
Fair	23.7		29.0

© 2024 Tucher EL et al. *JAMA Network Open.*

Good	37.1**		38.0**
Very Good	26.2***		20.2***
Excellent	5.7***		4.3***
Proxy Status, %		***	
Proxy Helped	13.2		9.2
Proxy Answered	4.0		3.3
Hospital Utilization, %		*	
No Utilization	87.4		88.2
1+ Visit	12.6		11.8
Lives Alone, %		***	
	34.3		30.7
Number of Days, N			
	104		102
**Plan Characteristics**			
Plan Type, %		***	
HMO	81.4		61.1
PPO	18.6		38.9
Plan Size, %			
Small (<750)	1.7		1.3
Medium (750-4,300,)	12.7		10.8
Large (>4,300)	85.6***		87.9***
SNP, %		***	
	22.2		9.4
Contract Start, %			
<2006	65.4		66.1
2006-2013	29.4		30.2
2014-2020	5.2***		3.7***
Max OOP, Mean		***	
	$5,016		$5,568
Change in Max OOP, Mean		***	
	-$119		-$39
Premium, Mean		***	
	$17		$10
Change in Premium, Mean		***	
	$0		-$3
Deductible, Mean			
	$44		$48
Change in Deductible, Mean			
	-$7		-$8
Enrollment, Mean		***	
	18,326		105,043
**County Characteristics**			
MA Penetration, %		***	
	47.2		43.6

SOURCE Authors' analysis of 2017-2021 MA CAHPS survey data and Medicare administrative enrollment data and publicly available MA plan benefit, enrollment, and crosswalk data. NOTES ESRD: End-Stage Renal Disease, HMO: Health Maintenance Organization, PPO: Preferred Provider Organization, OOP: Out-of-pocket; *p<0.05 **p<0.01 ***p<0.001

© 2024 Tucher EL et al. *JAMA Network Open.*

eTable 4. Unadjusted and Adjusted Impact of PHRB, SSBCI, or Both Benefit Adoption on Probability of Non-Response in the MA CAHPS, 2017-2021

PHRB							
	Treatment		Control		Unadj. DiD	Adj. DiD	95% CI
	Pre-Period	Post-Period	Pre-Period	Post-Period			
Non-Response, %	3.6	2.4	3.2	3.0	-1.0***	0.0	-0.7, 0.7
Sample, N	96,755	26,649	295,474	116,581	—	—	—
**SSBCI**							
	Treatment		Control		Unadj. DiD	Adj. DiD	95% CI
	Pre-Period	Post-Period	Pre-Period	Post-Period			
Non-Response, %	3.1	1.9	3.2	3.1	-1.1***	-0.5	-1.4, 0.4
Sample, N	51,298	13,897	224,351	114,363	—	—	—
**Both Benefits**							
	Treatment		Control		Unadj. DiD	Adj. DiD	95% CI
	Pre-Period	Post-Period	Pre-Period	Post-Period			
Non-Response, %	3.2	2.7	3.2	3.0	-0.3	0.2	-0.6, 1.1
Sample, N	49,164	17,332	225,132	122,621	—	—	—

SOURCE Authors' analysis of 2017-2021 MA CAHPS survey data and Medicare administrative enrollment data and publicly available MA plan benefit, enrollment, and crosswalk data. NOTES eTable 4 presents the unadjusted and adjusted difference-in-difference (DID) estimates of the impact of PHRB, SSBCI, or both benefits adoption on probability of non-response expressed as the odds of non-response across treatment and control plans. For all benefits, 2017-2019 is the pre-period and 2021 is the post-period. N represents the number of person-years in each treatment group. Fully adjusted models control for individual-level (sex, race/ethnicity, age, original reason for entitlement, dual eligibility, educational attainment, self-rated physical and mental health, receipt of proxy assistance, self-reported hospitalization utilization, living alone, self-reported chronic conditions, census region, inpatient admissions, nursing home admissions, home health visits, chronic conditions, and number of days in 2021 an enrollee was in the plan), plan-level factors (plan type, plan size, SNP status, contract start year, max out of pocket (OOP), premium, deducible, and change in max OOP, premium, and deductible from the prior year, and plan age), and county MA penetration rate. ***p<0.001, **p<0.01, *p<0.05.

© 2024 Tucher EL et al. *JAMA Network Open.*

eTable 5. Unadjusted and Adjusted Impact of PHRB Adoption on Plan Rating, 2019

	**PHRB**						
	Treatment		Control		Unadj. DiD	Adj. DiD	95% CI
	Pre-Period	Post-Period	Pre-Period	Post-Period			
Plan Rating, Mean	8.63	8.66	8.68	8.75	-0.04	-0.03	-0.10, 0.03
Sample, N	51,062	25,810	241,492	121,049	—	—	—

SOURCE Authors' analysis of 2017-2021 MA CAHPS survey data and Medicare administrative enrollment data and publicly available MA plan benefit, enrollment, and crosswalk dat. NOTES eTable 5 presents the unadjusted and adjusted difference-in-difference (DID) estimates of the impact of PHRB adoption on individual plan rating expressed as the average plan-rating across treatment and control plans. For all benefits, 2017-2018 is the pre-period and 2019 is the post-period. N represents the number of person-years in each treatment group. Fully adjusted models control for individual-level (sex, race/ethnicity, age, original reason for entitlement, dual eligibility, educational attainment, self-rated physical and mental health, receipt of proxy assistance, self-reported hospitalization utilization, living alone, self-reported chronic conditions, census region, inpatient admissions, nursing home admissions, home health visits, chronic conditions, and number of days in 2021 an enrollee was in the plan), plan-level factors (plan type, plan size, SNP status, contract start year, max out of pocket (OOP), premium, deducible, and change in max OOP, premium, and deductible from the prior year, and plan age), and county MA penetration rate. ***p<0.001, **p<0.01, *p<0.05.

© 2024 Tucher EL et al. *JAMA Network Open.*

eTable 6. Unadjusted and Adjusted Impact of PHRB, SSBCI, or Both Benefit Adoption on Plan Rating, 2017-2021

PHRB

	Treatment		Control		Unadj. DiD	Adj. DiD	95% CI
	Pre-Period	Post-Period	Pre-Period	Post-Period			
Plan Rating, Mean	8.69	8.75	8.68	8.77	-0.03	-0.04	-0.10, 0.01
Sample, N	108,729	50,942	222,242	199,239	—	—	—

SSBCI

	Treatment		Control		Unadj. DiD	Adj. DiD	95% CI
	Pre-Period	Post-Period	Pre-Period	Post-Period			
Plan Rating, Mean	8.80	9.00	8.69	8.80	0.09	0.04	-0.06, 0.13
Sample, N	89,081	13,695	275,438	92,670	—	—	—

Both Benefits

	Treatment		Control		Unadj. DiD	Adj. DiD	95% CI
	Pre-Period	Post-Period	Pre-Period	Post-Period			
Plan Rating, Mean	8.79	8.92	8.70	8.77	0.06	0.02	-0.07, 0.12
Sample, N	48,364	18,060	245,247	102,673	—	—	—

SOURCE Authors' analysis of 2017-2021 MA CAHPS survey data and Medicare administrative enrollment data and publicly available MA plan benefit, enrollment, and crosswalk data. NOTES eTable 6 presents the unadjusted and adjusted difference-in-difference (DID) estimates of the impact of PHRB, SSBCI, or both benefits adoption on individual plan rating expressed as the average plan-rating across treatment and control plans. N represents the number of person-years in each treatment group. Fully adjusted models control for individual-level (sex, race/ethnicity, age, original reason for entitlement, dual eligibility, educational attainment, self-rated physical and mental health, receipt of proxy assistance, self-reported hospitalization utilization, living alone, self-reported chronic conditions, census region, inpatient admissions, nursing home admissions, home health visits, chronic conditions, and number of days in 2021 an enrollee was in the plan), plan-level factors (plan type, plan size, SNP status, contract start year, max out of pocket (OOP), premium, deducible, and change in max OOP, premium, and deductible from the prior year, and plan age), and county MA penetration rate. ***$p<0.001$, **$p<0.01$, *$p<0.05$.

© 2024 Tucher EL et al. *JAMA Network Open.*

eTable 7. Unadjusted and Adjusted Impact of PHRB, SSBCI, or Both Benefit Adoption on Plan Rating for Plans that Adopted Two or More Benefits, 2021

2+ PHRB Benefits

	Treatment		Control		Unadj. DiD	Adj. DiD	95% CI
	Pre-Period	Post-Period	Pre-Period	Post-Period			
Plan Rating, Mean	8.64	8.93	8.71	8.87	0.13	-0.14	-0.37, 0.08
Sample, N	2,004	2,115	4,102	8,802	—	—	—

2+ SSBCI Benefits

	Treatment		Control		Unadj. DiD	Adj. DiD	95% CI
	Pre-Period	Post-Period	Pre-Period	Post-Period			
Plan Rating, Mean	8.66	9.14	8.64	8.84	0.30	[a]	[a]
Sample, N	2,302	1,170	51,556	26,061	—	—	—

2+ PHRB and 2+ SSBCI (Both) Benefits

	Treatment		Control		Unadj. DiD	Adj. DiD	95% CI
	Pre-Period	Post-Period	Pre-Period	Post-Period			
Plan Rating, Mean	8.50	9.06	8.77	8.81	0.53*	0.45	-0.08, 0.98
Sample, N	4,512	3,550	131	79	—	—	—

SOURCE Authors' analysis of 2017-2021 MA CAHPS survey data and Medicare administrative enrollment data and publicly available MA plan benefit, enrollment, and crosswalk data. NOTES eTable 7 presents the unadjusted and adjusted difference-in-difference (DID) estimates of the impact of PHRB, SSBCI, or both benefits adoption on individual plan rating expressed as the average plan-rating across treatment and control plans for plans that adopted more than one benefit. N represents the number of person-years in each treatment group. Fully adjusted models control for individual-level (sex, race/ethnicity, age, original reason for entitlement, dual eligibility, educational attainment, self-rated physical and mental health, receipt of proxy assistance, self-reported hospitalization utilization, living alone, self-reported chronic conditions, census region, inpatient admissions, nursing home admissions, home health visits, chronic conditions, and number of days in 2021 an enrollee was in the plan), plan-level factors (plan type, plan size, SNP status, contract start year, max out of pocket (OOP), premium, deducible, and change in max OOP, premium, and deductible from the prior year, and plan age), and county MA penetration rate. All analyses apply survey weights. ***p<0.001, **p<0.01, *p<0.05. [a]Enrollees in plans that adopted two or more SSBCI benefits are removed from the analysis because the trends were not graphically or statistically (p=0.02) parallel prior to adoption in 2021.

© 2024 Tucher EL et al. *JAMA Network Open.*

eTable 8. Unadjusted and Adjusted Impact of PHRB, SSBCI, or Both Benefit Adoption on Health Utilization and Self-Reported Health, 2021

	Treatment		Control		Unadj. DiD	Adj. DiD	95% CI
**PHRB Benefits**							
	Pre-Period	Post-Period	Pre-Period	Post-Period			
One Plus Hospital Stay, %	12.7	9.0	11.6	9.9	-2.0	-0.04	-2.8, 2.7
Sample, N	16,910	9,682	63,019	47,065	—	—	—
Care As Soon as Needed, Mean	3.66	3.65	3.61	3.62	-0.02	0.09	0.00, 0.18
Sample, N	5,479	2,577	17,682	9,985	—	—	—
Number of Doctors Visits, Mean	2.36	2.16	2.42	2.15	0.06	0.12	-0.01, 0.26
Sample, N	16,238	9,387	58,311	45,470	—	—	—
Self-Rated Health, Mean	1.86	1.84	1.83	1.78	0.03	0.01	-0.04, 0.06
Sample, N	16,541	9,563	59,567	46,176	—	—	—
**SSBCI Benefits**							
	Treatment		Control		Unadj. DiD	Adj. DiD	95% CI
	Pre-Period	Post-Period	Pre-Period	Post-Period			
One Plus Hospital Stay, %	12.8	10.5	11.9	9.4	0.3	-0.9	-2.8, 1.1
Sample, N	31,179	11,204	150,067	75,537	—	—	—
Care As Soon as Needed, Mean	3.61	3.58	3.60	3.59	-0.02	0.04	-0.06, 0.14
Sample, N	10,950	3,358	44,788	16,019	—	—	—
Number of Doctors Visits, Mean	2.37	2.09	2.41	2.09	0.03	0.02	-0.09, 0.13
Sample, N	29,572	10,659	143,016	72,982	—	—	—
Self-Rated Health, Mean	2.03	2.04	1.84	1.81	[a]	0.01	-0.05, 0.06
Sample, N	30,380	10,968	146,392	74,072	—	—	—
**Both Benefits**							
	Treatment		Control		Unadj. DiD	Adj. DiD	95% CI
	Pre-Period	Post-Period	Pre-Period	Post-Period			
One Plus Hospital Stay, %	12.3	9.4	11.5	9.7	-1.1	-1.0	-4.2, 2.2
Sample, N	10,656	4,450	75,782	37,899	—	—	—
Care As Soon as Needed, Mean	3.52	3.57	3.59	3.57	0.07	0.04	-0.16, 0.24
Sample, N	3,441	1,143	20,497	7,862	—	—	—

© 2024 Tucher EL et al. *JAMA Network Open.*

Number of Doctors Visits, Mean	2.40	2.07	2.39	2.06	0.01	-0.09	-0.26, 0.08
Sample, N	10,298	4,303	72,635	36,630	—	—	—
Self-Rated Health, Mean	2.06	1.97	1.82	1.80	-0.07	-0.04	-0.11, 0.03
Sample, N	10,401	4,406	73,894	37,338	—	—	—

SOURCE Authors' analysis of 2017-2021 MA CAHPS survey data and Medicare administrative enrollment data and publicly available MA plan benefit, enrollment, and crosswalk data. NOTES eTable 8 presents the unadjusted and adjusted difference-in-difference (DID) estimates of the impact of PHRB, SSBCI, or both benefits adoption on individual health utilization metrics and self-rated health expressed as the average health utilization metrics and self-rated health across treatment and control plans for plans that adopted more than one benefit. N represents the number of person-years in each treatment group. Fully adjusted models control for individual-level (sex, race/ethnicity, age, original reason for entitlement, dual eligibility, educational attainment, self-rated mental health, receipt of proxy assistance, self-reported hospitalization utilization, living alone, self-reported chronic conditions, census region, inpatient admissions, nursing home admissions, home health visits, chronic conditions, and number of days in 2021 an enrollee was in the plan), plan-level factors (plan type, plan size, SNP status, contract start year, max out of pocket (OOP), premium, deducible, and change in max OOP, premium, and deductible from the prior year, and plan age), and county MA penetration rate. All analyses apply survey weights. ***p<0.001, **p<0.01, *p<0.05. [a]Enrollees in plans that adopted both are removed from the unadjusted analysis because the trends were not graphically or statistically (p=0.02) parallel prior to adoption in 2021 in fully adjusted models.

© 2024 Tucher EL et al. *JAMA Network Open.*

eFigure 2. Adjusted Event Study for Primarily Health Related Benefit (PHRB) Adoption, 2019-2021

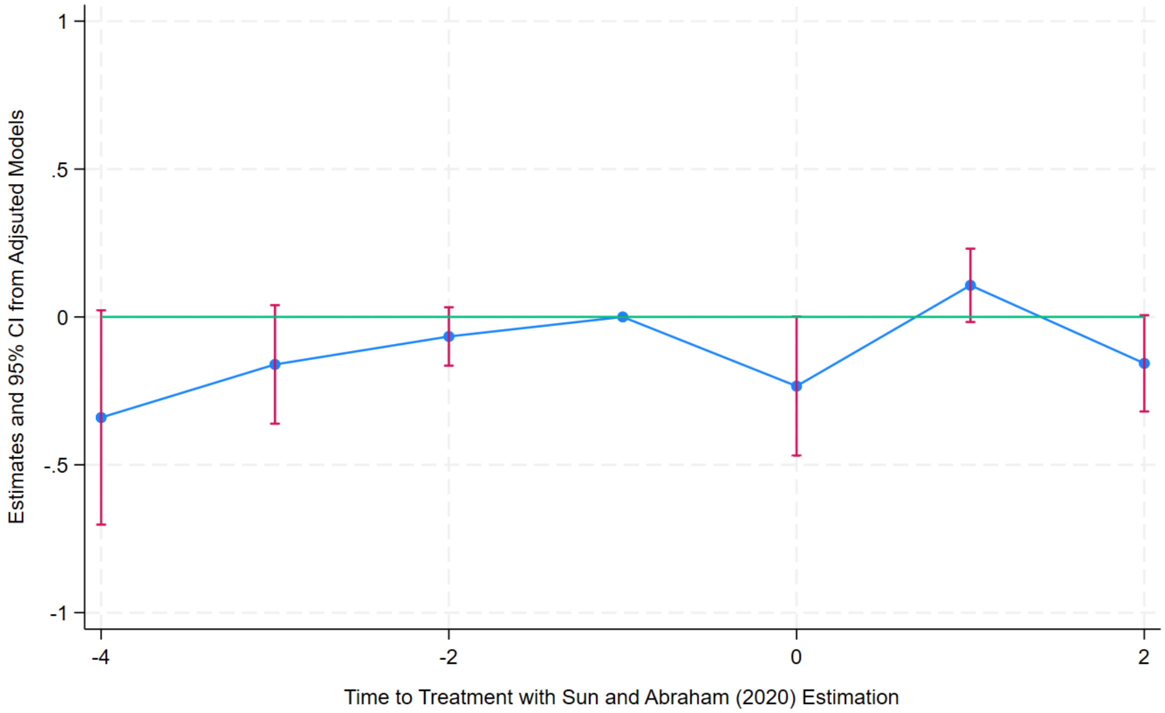

SOURCE Authors' analysis of 2017-2021 MA CAHPS survey data and Medicare administrative enrollment data and publicly available MA plan benefit, enrollment, and crosswalk data. NOTES Estimates reflect outcomes from an adjusted event study for plans that adopted a primarily health related benefit (PHRB) in 2019, 2020, or 2021 that did not adopt a SSBCI at any point; 2020 CAHPS data is unavailable and therefore is not shown. We removed plans that adopted the PHRBs in 2019 only, 2019 and 2020 only, 2019 and 2021 only and plans that adopted the SSBCIs in 2021 only (634 plans, 37% of the sample). Fully adjusted models control for individual-level (sex, race/ethnicity, age, original reason for entitlement, dual eligibility, educational attainment, self-rated mental health, receipt of proxy assistance, self-reported hospitalization utilization, living alone, self-reported chronic conditions, census region, inpatient admissions, nursing home admissions, home health visits, chronic conditions, and number of days in 2021 an enrollee was in the plan), plan-level factors (plan type, plan size, SNP status, contract start year, max out of pocket [OOP], premium, deducible, and change in max OOP, premium, and deductible from the prior year, and plan age), and county MA penetration rate. All analyses apply survey weights.

eFigure 3. Unadjusted Event Study for Primarily Health Related Benefit (PHRB) Adoption, 2019-2021

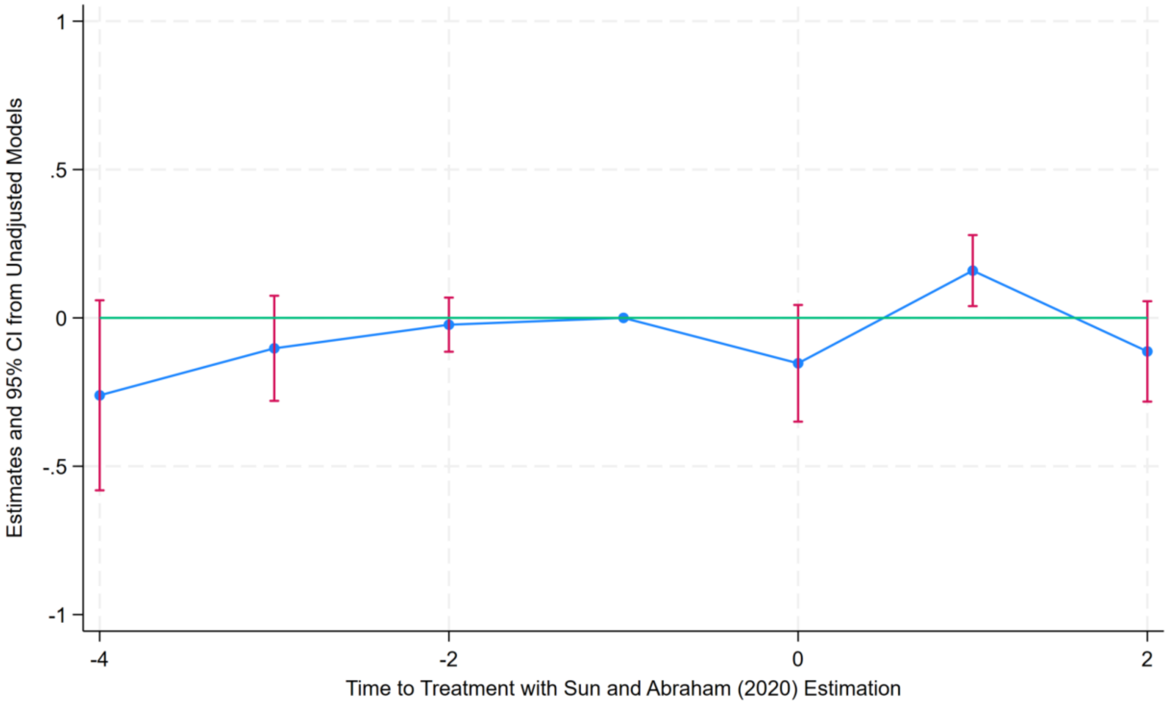

SOURCE Authors' analysis of 2017-2021 MA CAHPS survey data and Medicare administrative enrollment data and publicly available MA plan benefit, enrollment, and crosswalk data. NOTES Estimates reflect outcomes from an unadjusted event study for plans that adopted a primarily health related benefit (PHRB) in 2019, 2020, or 2021 that did not adopt a SSBCI at any point; 2020 CAHPS data is unavailable and therefore is not shown. We removed plans that adopted the PHRBs in 2019 only, 2019 and 2020 only, 2019 and 2021 only and plans that adopted the SSBCIs in 2021 only (634 plans, 37% of the sample). All analyses apply survey weights.

© 2024 Tucher EL et al. *JAMA Network Open.*

eFigure 4. Adjusted Event Study for Special Supplemental Benefits for the Chronically Ill (SSBCI) Adoption, 2020-2021

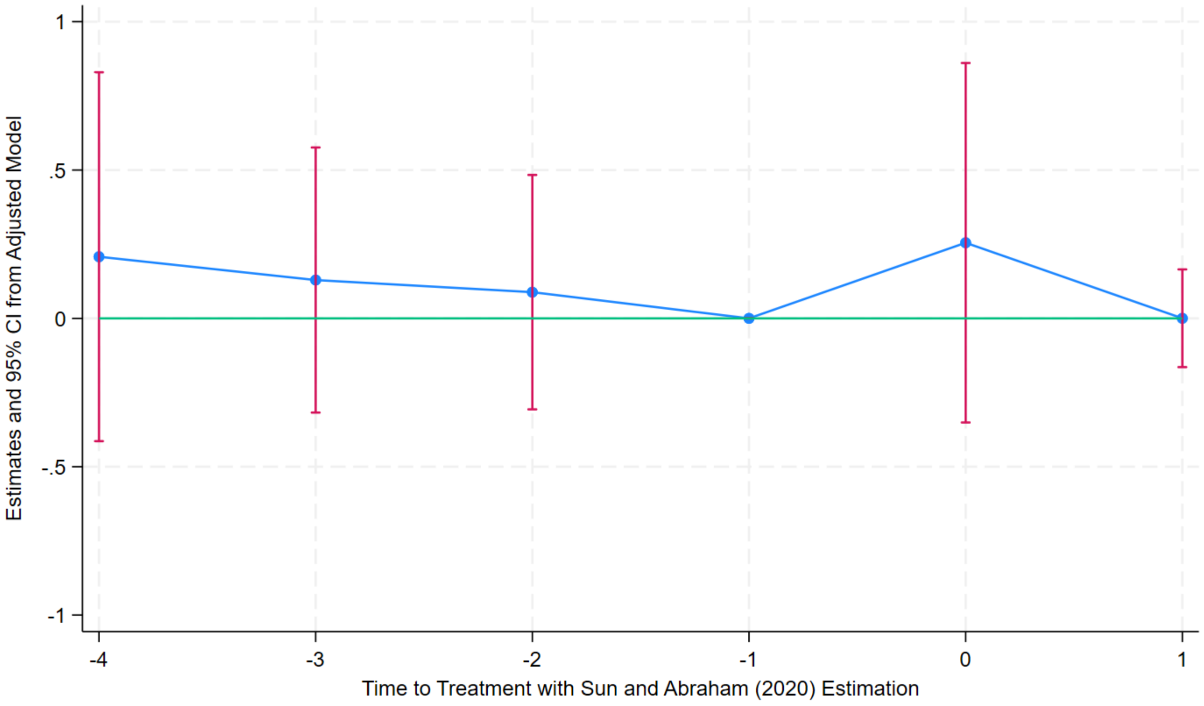

SOURCE Authors' analysis of 2017-2021 MA CAHPS survey data and Medicare administrative enrollment data and publicly available MA plan benefit, enrollment, and crosswalk data. NOTES Estimates reflect outcomes from an adjusted event study for plans that adopted a Special Supplemental Benefits for the Chronically Ill (SSBCI) in 2020 or 2021 that did not adopt a PHRB at any point; 2020 CAHPS data is unavailable and therefore is not shown. We removed plans that adopted the PHRBs in 2019 only, 2019 and 2020 only, 2019 and 2021 only and plans that adopted the SSBCIs in 2021 only (634 plans, 37% of the sample). Fully adjusted models control for individual-level (sex, race/ethnicity, age, original reason for entitlement, dual eligibility, educational attainment, self-rated mental health, receipt of proxy assistance, self-reported hospitalization utilization, living alone, self-reported chronic conditions, census region, inpatient admissions, nursing home admissions, home health visits, chronic conditions, and number of days in 2021 an enrollee was in the plan), plan-level factors (plan type, plan size, SNP status, contract start year, max OOP, premium, deducible, and change in max OOP, premium, and deductible from the prior year, and plan age), and county MA penetration rate. All analyses apply survey weights.

© 2024 Tucher EL et al. *JAMA Network Open.*

eFigure 5. Unadjusted Event Study for Special Supplemental Benefits for the Chronically Ill (SSBCI) Adoption, 2020-2021

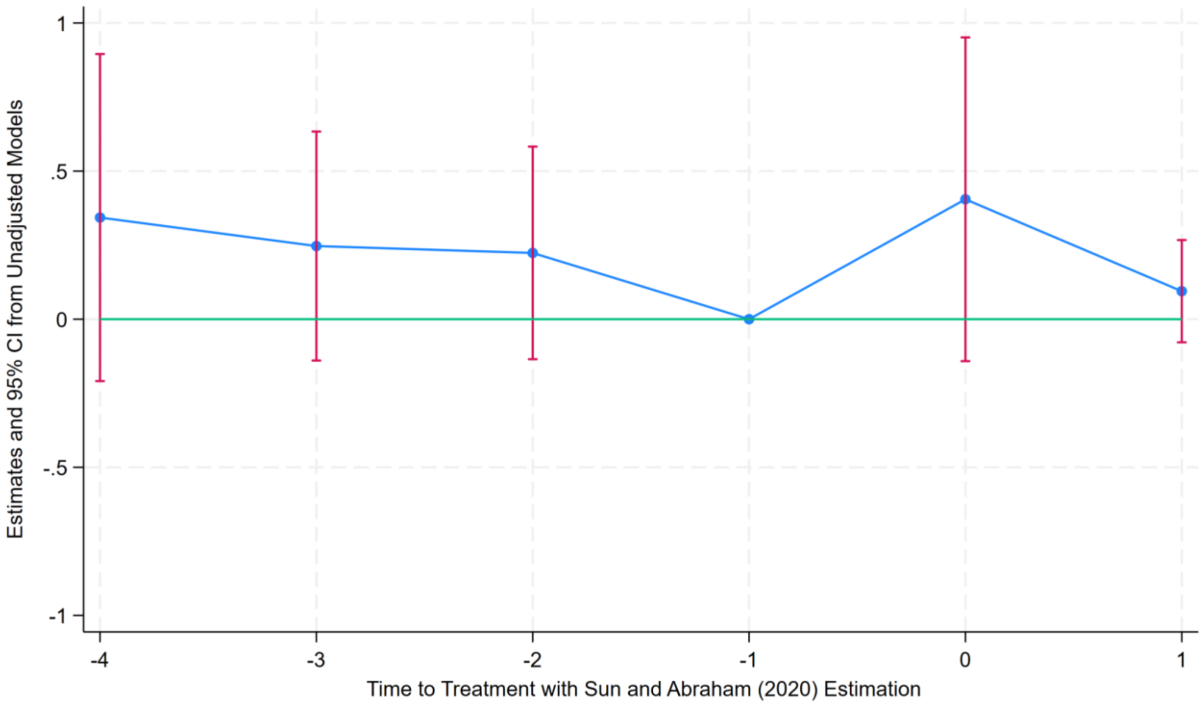

SOURCE Authors' analysis of 2017-2021 MA CAHPS survey data and Medicare administrative enrollment data and publicly available MA plan benefit, enrollment, and crosswalk data. NOTES Estimates reflect outcomes from an adjusted event study for plans that adopted a Special Supplemental Benefits for the Chronically Ill (SSBCI) in 2020 or 2021 that did not adopt a PHRB at any point; 2020 CAHPS data is unavailable and therefore is not shown. We removed plans that adopted the PHRBs in 2019 only, 2019 and 2020 only, 2019 and 2021 only and plans that adopted the SSBCIs in 2021 only (634 plans, 37% of the sample). All analyses apply survey weights.

© 2024 Tucher EL et al. *JAMA Network Open.*

eFigure 6. Adjusted Event Study for Plans that Adopted Both Benefits, 2020-2021

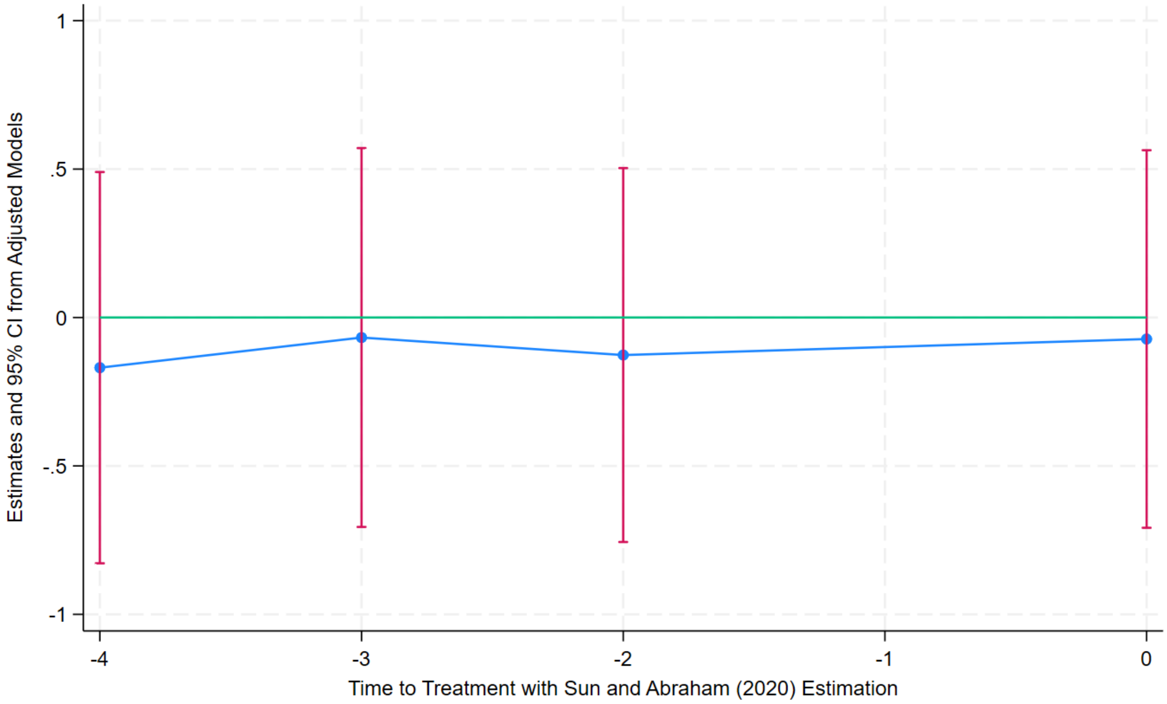

SOURCE Authors' analysis of 2017-2021 MA CAHPS survey data and Medicare administrative enrollment data and publicly available MA plan benefit, enrollment, and crosswalk data. NOTES Estimates reflect outcomes from an adjusted event study for plans that adopted a PHRB and SSBCI in 2020 or 2021 that did not previously adopt either; 2020 CAHPS data is unavailable and therefore is not shown. We removed plans that adopted the PHRBs in 2019 only, 2019 and 2020 only, 2019 and 2021 only and plans that adopted the SSBCIs in 2021 only (634 plans, 37% of the sample). Fully adjusted models control for individual-level (sex, race/ethnicity, age, original reason for entitlement, dual eligibility, educational attainment, self-rated mental health, receipt of proxy assistance, self-reported hospitalization utilization, living alone, self-reported chronic conditions, census region, inpatient admissions, nursing home admissions, home health visits, chronic conditions, and number of days in 2021 an enrollee was in the plan), plan-level factors (plan type, plan size, SNP status, contract start year, max OOP, premium, deducible, and change in max OOP, premium, and deductible from the prior year, and plan age), and county MA penetration rate. All analyses apply survey weights.

© 2024 Tucher EL et al. *JAMA Network Open.*

eFigure 7. Unadjusted Event Study for Plans that Adopted Both Benefits, 2020-2021

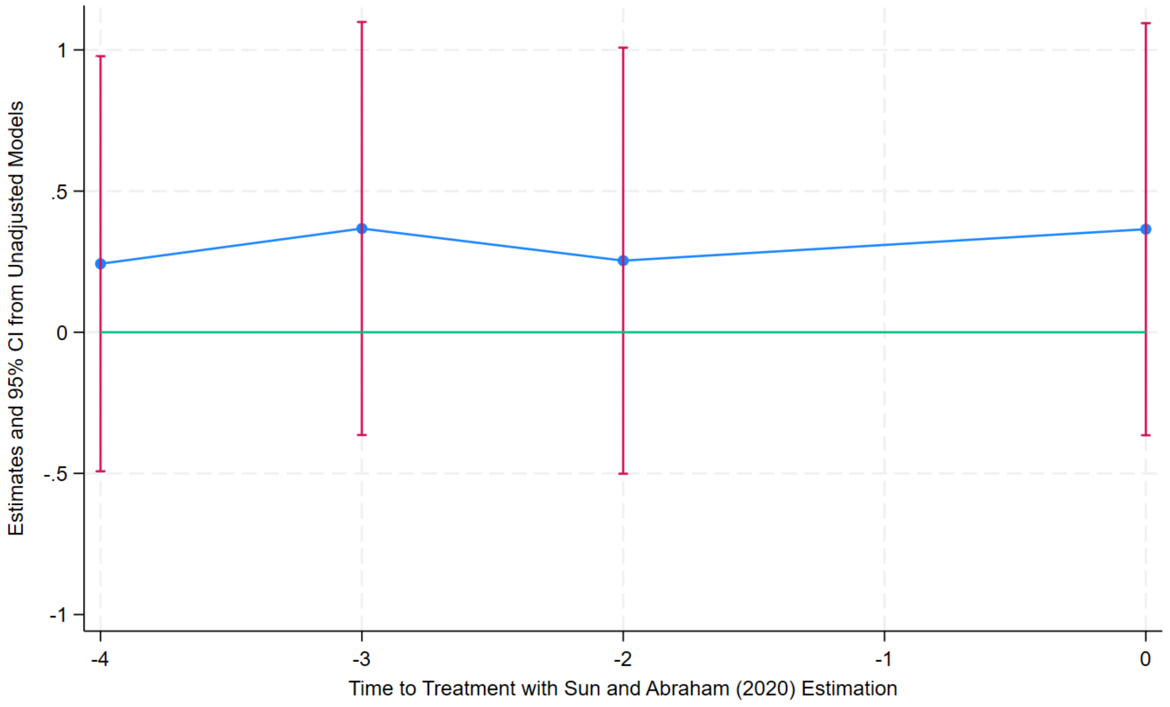

SOURCE Authors' analysis of 2017-2021 MA CAHPS survey data and Medicare administrative enrollment data and publicly available MA plan benefit, enrollment, and crosswalk data. NOTES Estimates reflect outcomes from an adjusted event study for plans that adopted a PHRB and SSBCI in 2020 or 2021 that did not previously adopt either; 2020 CAHPS data is unavailable and therefore is not shown. We removed plans that adopted the PHRBs in 2019 only, 2019 and 2020 only, 2019 and 2021 only and plans that adopted the SSBCIs in 2021 only (634 plans, 37% of the sample). All analyses apply survey weights.

© 2024 Tucher EL et al. *JAMA Network Open.*

eTable 9. Rate of Extra Benefit Availability Due to Health Condition Across Benefit Cohort, 2017-2021

	Health plan offering benefit due to health condition			
	2017	2018	2019	2021
	**PHRB Cohort**			
Treatment				
Sample, N	2,929	3,529	2,870	4,955
Yes, %	7.0	7.2	7.8	9.1
No, %	93.0	92.8	92.2	90.9
Control				
Sample, N	25,635	25,061	20,754	55,712
Yes	6.5	6.1	6.8	6.6
No	93.5	93.9	93.2	93.4
	**SSBCI Cohort**			
Treatment				
Sample, N	6,811	6,000	4,312	6,873
Yes, %	8.2	8.6	11.0	11.2
No, %	91.8	91.4	89.0	88.8
Control				
Sample, N	55,471	57,939	51,318	84,174
Yes, %	6.6	6.9	7.9	7.2
No, %	93.4	93.1	92.1	93.8
	**Adopted Both Benefits Cohort**			
Treatment				
Sample, N	3,017	2,610	2,392	3,966
Yes, %	8.7	10.4	9.0	11.0
No, %	91.3	89.6	91.0	89.0
Control				
Sample, N	26,356	27,291	25,127	48,714
Yes, %	6.3	6.7	6.8	7.5
No, %	93.7	93.3	93.2	92.5

SOURCE Authors' analysis of 2017-2021 MA CAHPS survey data and Medicare administrative enrollment data and publicly available MA plan benefit, enrollment, and crosswalk data. NOTES Question from the MA CAHPS Survey: Your health plan benefits are the types of health care and services you can get under the plan. In the last 6 months, [MA-Only] did your health plan offer you extra benefits because you have a health condition (like high blood pressure)?

© 2024 Tucher EL et al. *JAMA Network Open.*

eTable 10. Relationship Between Supplemental Benefit Adoption and Enrollee Receipt of Extra Benefit Due to Health Condition, 2017-2021

Extra Benefit Availability Due to Health Condition		
	Coefficient [95% CI]	
PHRB	Unadjusted	Adjusted
2017	0.01 [-0.01, 0.03]	0.02 [0.0, 0.04]
2018	0.02 [0.0, 0.4]	0.02 [0.0, 0.05]
2019	0.02 [-0.01, 0.04]	-0.03 [-0.05, -0.01]**
2021	0.03 [0.01, 0.05] ***	0.01 [-0.01, 0.03]
SSBCI	Unadjusted	Adjusted
2017	0.02 [0.01, 0.04]***	-0.01 [-0.02, 0.01]
2018	0.02 [0.01, 0.03]***	0.00 [-0.01, 0.01]
2019	0.03 [0.02, 0.04]***	0.00 [-0.01, 0.01]
2021	0.04 [0.03, 0.05]***	0.01 [-0.01, 0.02]
Both	Unadjusted	Adjusted
2017	0.04 [0.01, 0.07]*	0.00 [-0.03, 0.03]
2018	0.04 [0.02, 0.07]**	0.00 [-0.02, 0.03]
2019	0.03 [0.00, 0.05]*	0.00 [-0.03, 0.02]
2021	0.03 [0.02, 0.05]***	0.00 [-0.01, 0.02]

SOURCE Authors' analysis of 2017-2021 MA CAHPS survey data and Medicare administrative enrollment data and publicly available MA plan benefit, enrollment, and crosswalk data. NOTES eTable 10 presents a linear regression of the relationship between supplemental benefit adoption and an enrollee's self-reported receipt of receiving an additional benefit from their health plan to help manage their chronic condition. ***p<0.001, **p<0.01, *p<0.05.

© 2024 Tucher EL et al. *JAMA Network Open.*
