# Supplementary material for: New Supplemental Benefits and Plan Ratings Among Medicare Advantage Enrollees
Source: JAMA Netw Open. 2024 Jun 5;7(6):e2415058. doi: 10.1001/jamanetworkopen.2024.15058 (PMC11154155; doi:10.1001/jamanetworkopen.2024.15058)
Supplement: Supplement 2. — Data Sharing Statement [file jamanetwopen-e2415058-s002.pdf]

Data Sharing Statement

Tucher. New Supplemental Benefits and Plan Ratings Among Medicare Advantage Enrollees. *JAMA Netw Open*. Published June 05, 2024. doi:10.1001/jamanetworkopen.2024.15058

Data
Data available: No

Additional Information
Explanation for why data not available: The data is accessible to the research team through a data use agreement between CMS and the research team at Brown University. We are not able to provide access to these data.
